# 22566 Identifying metabolic mechanisms linking prenatal acetaminophen exposure to childhood attention-deficit hyperactivity disorder

**DOI:** 10.1017/cts.2021.707

**Published:** 2021-03-30

**Authors:** Neha S. Anand, Xiaobin Wang

**Affiliations:** 1 Johns Hopkins School of Medicine; 2 Johns Hopkins Bloomberg School of Public Health

## Abstract

ABSTRACT IMPACT: This study has implications for understanding early developmental mechanisms of ADHD and for guidelines regarding safe use of acetaminophen during pregnancy. OBJECTIVES/GOALS: Prenatal acetaminophen exposure has been associated with childhood attention-deficit hyperactivity disorder (ADHD), but the underlying mechanism is unknown. This prospective birth cohort study aims to identify linkages between specific metabolites in umbilical cord plasma and the association of prenatal acetaminophen exposure and ADHD. METHODS/STUDY POPULATION: The sample was a subset of the Boston Birth Cohort that included 583 mother-newborn dyads followed at Boston Medical Center from 1998 to 2018. Metabolites were measured from cord plasma collected at birth. Based on existing literature, the analyses focused on candidate metabolites involved in neuroendocrine, inflammation, and oxidative stress pathways. The outcome was physician-diagnosed ADHD between the ages of 3 and 16 years. Exploratory analyses and multiple logistic regressions were used to examine the association of these candidate metabolites with both unmetabolized cord plasma acetaminophen levels and with incident risk of ADHD, adjusting for covariates of maternal and child characteristics. RESULTS/ANTICIPATED RESULTS: Of the 583 children, 257 had ADHD and 326 had neurotypical development. Two promising results have been found thus far. 5-methoxytryptophol (5-MTX), a neuroendocrine molecule which also has antioxidant and immunomodulatory properties, was inversely associated with acetaminophen and ADHD risk. For children below the median cord 5-MTX level, the odds of ADHD were 3.29 (95% CI [1.56, 7.16], p=0.002) for the third tertile of acetaminophen compared to the first tertile. This association attenuated among those above the median 5-MTX level: 2.23 (95% CI [0.98, 5.21], p=0.059), suggesting a protective effect. Tryptophan, an essential amino acid and precursor of serotonin, was positively associated with acetaminophen and ADHD. Next steps include mediation analysis with tryptophan and analyses for other metabolites. DISCUSSION/SIGNIFICANCE OF FINDINGS: This study identifies cord plasma metabolites as possible modifiers or mediators linking prenatal acetaminophen exposure and childhood ADHD, which may offer insight into a mechanistic pathway. The study findings have implications for FDA, clinical, and public health guidelines regarding safe use of acetaminophen during pregnancy.

